# Mental Health Home Intensive Care during Spanish national lockdown due to COVID-19 Pandemic

**DOI:** 10.1192/j.eurpsy.2023.880

**Published:** 2023-07-19

**Authors:** L. Alba, L. Alonso, H. Pérez, S. Saez, C. Coll, S. Palma, S. Ortiz

**Affiliations:** Intensive Home Care Service of the Baix Llobregat Center area, Parc Sanitari Sant Joan de Déu, Barcelona, Spain

## Abstract

**Introduction:**

The SARS-CoV-2 pandemic situation forced the Spanish Government to declare a home confinement that was prolonged for three months. The Health System had to focus almost entirely on the treatment of patients with Covid-19 infection and vulnerable populations such as people with severe mental illness were overlooked. In this context, mental health home care as an alternative to hospitalization became a first-line approachment for patients with an acute mental health disorder.

**Objectives:**

The aim of this study is to describe the professional practice and the patients characteristics attended by a mental health team in Catalonia during the home confinement due to the SARS-CoV-2 pandemic.

**Methods:**

This study includes the period between March 15, 2020 and June 21, 2020. Sociodemographic, clinical and team functioning variables were described. The patient data was obtained from medical history whereas the rest of information was collected through the creation of a database by the members of the assistencial team.

**Results:**

Team’s structure was changed with the creation of two mirror teams. Psychological attention was expanded and the weekend coverage was reorganized.During that period 40 patients were treated with a mean age of 47 years. There were no differences regarding the gender (50% were men and 50% were women). 87% of the patients lived with their family. 75% of referrals were made by the hospital and 90% were referred to community services upon discharge. Regarding diagnoses, 57.5% of the patients were diagnosed with a psychotic disorder (10% being reactive to Covid-19). A total of 482 visits were made, of which 51% were conducted in a telematic form. The mean time between the referral and the admission was 37.98h. The team accepted 97% of the referred patients with an occupancy rate of 112.4%.

**Conclusions:**

The re-organisation of a home treatment team during the domiciliary lockdown allowed to increase the occupancy rate and offered a rapid response to patients, avoiding the need of conventional hospitalization and providing a proper care plan.

**Disclosure of Interest:**

None Declared

